# Pay-It-Forward 23-Valent Pneumococcal Polysaccharide Vaccination Among Older Adults: Protocol for a Randomized Controlled Trial

**DOI:** 10.2196/70246

**Published:** 2025-09-08

**Authors:** Jiao Qin, Liangjia Wei, Chunxing Tao, Jinfeng He, Dan Wu, Zhifeng Lin, Ting Huang, Shiyu Qin, Qiuqian Su, Yanxiao Gao, Shuiming Chen, Ganqin Wang, Xianyan Tang, Li Ye, Hao Liang, Chuanyi Ning, Weiming Tang, Joseph D Tucker, Bingyu Liang

**Affiliations:** 1 Guangxi Key Laboratory of AIDS Prevention and Treatment School of Public Health & Life Science Institute Guangxi Medical University Nanning China; 2 Department of Social Medicine and Health Education School of Public Health Nanjing Medical University Nanjing China; 3 Shenzhen Institute of Advanced Technology Chinese Academy of Sciences Shenzhen China; 4 Jinzhou Community Health Center Nanning China; 5 West of Panlong Community Health Center The Second People's Hospital of Nanning Nanning China; 6 Dermatology Hospital of Southern Medical University Guangzhou China; 7 University of North Carolina at Chapel Hill, Project-China Guangzhou China; 8 London School of Hygiene and Tropical Medicine Clinical Research Department London United Kingdom; 9 Department of Medicine University of North Carolina at Chapel-Hill Chapel Hill, NC United States

**Keywords:** randomized controlled trial, 23-valent pneumococcal polysaccharide vaccine, pay-it-forward, older adults, China

## Abstract

**Background:**

The 23-valent pneumococcal polysaccharide vaccine reduces the risk of pneumonia among adults by 38% to 46%. However, only a few older adults in resource-limited areas of China have received the pneumococcal vaccination. Pay-it-forward is a social innovation that offers participants free or subsidized health services and a community-engaged message, with an opportunity to donate to support subsequent recipients.

**Objective:**

This study aims to assess the effectiveness and cost-effectiveness of the pay-it-forward intervention in encouraging the uptake of the 23-valent pneumococcal polysaccharide vaccine in adults aged ≥60 years.

**Methods:**

A 2-arm, parallel randomized controlled trial will be conducted in 4 community health centers in Nanning city, Guangxi province, China. We will use a block randomization design. A total of 204 older adults will be randomly allocated in a 1:1 ratio to either the pay-it-forward group or the standard-of-care group. Each participant will complete a web-based questionnaire. The standard-of-care group will be required to pay for the vaccine themselves. In contrast, the pay-it-forward group will receive a 150 RMB (US $20.7) vaccination subsidy, postcards, and the opportunity to donate. The participants in both groups will be followed up in the second and fourth weeks after enrollment. The primary outcome will be uptake of the 23-valent pneumococcal polysaccharide vaccine, as determined by administrative data. Secondary outcomes include costs, pneumococcal vaccination knowledge, attitudes toward the vaccine, perceptions of gratitude, incidence of adverse reactions and adverse events, and the likelihood of recommending pneumococcal vaccination to others.

**Results:**

Participant recruitment and follow-up were conducted from January 2024 to September 2024. A total of 220 participants were enrolled. Finalized results are expected in June 2026.

**Conclusions:**

This study will provide evidence on the effectiveness and economic costs of the pay-it-forward strategy for pneumonia vaccination among older adults. The findings could have implications for vaccination policy and offer a new approach for increasing vaccination in resource-limited areas.

**Trial Registration:**

Chinese Clinical Trial Registry ChiCTR2400079410; https://www.chictr.org.cn/showprojEN.html?proj=213999

**International Registered Report Identifier (IRRID):**

DERR1-10.2196/70246

## Introduction

### Background

*Streptococcuspneumoniae* poses a significant threat to global health, contributing substantially to morbidity and mortality [1,2]. Furthermore, it is recognized as the most prevalent bacterial agent that causes community-acquired pneumonia, a condition associated with increased morbidity, mortality, and economic burden, particularly among individuals aged >65 years [3,4]. Globally, pneumococcal complications were present in 90% of older adults (aged ≥65 years) from 2005 to 2014 [5], with *S* *pneumoniae*–associated lower respiratory infections responsible for 500,000 deaths (45.74% of global pneumococcal deaths) in those aged ≥70 years in 2016 [6]. The estimated incidence of community-acquired pneumonia among individuals aged ≥60 years in China was 34.68 per 1000 person‐years in 2016 [7]. The in-hospital mortality rate for patients aged ≥65 years with community-acquired pneumonia from January to December 2014 was 5.7% [8]. The disease burden of influenza virus infections is equally severe as pneumonia among older adults. A population-based study demonstrated that adults aged 65 to 74 years experienced influenza-associated excess respiratory mortality rates ranging from 2.9 to 44 per 100,000, while those aged ≥75 years faced even higher rates, ranging from 17.9 to 223.5 per 100,000 [9]. Influenza viruses typically promote secondary pneumococcal infections [10], and their coinfection significantly increases the risk of hospitalization, serious illness, and death [11]. This underscores the need for pneumococcal and influenza vaccines to prevent community-acquired pneumonia and influenza.

The World Health Organization and the Chinese Center for Disease Control and Prevention recommend a 23-valent pneumococcal polysaccharide vaccine (PPSV-23) for adults aged ≥60 years [12]. The PPSV-23 has a 30.9% efficacy in preventing community-acquired pneumonia [13] and offers a 70% protective efficacy against invasive pneumococcal diseases [14]. Furthermore, the vaccine’s protective benefits are known to be durable for up to 5 years [15]. Despite these advantages, the Chinese government has yet to incorporate PPSV-23 into its free routine immunization program for older adults. Consequently, pneumococcal vaccination remains an out-of-pocket service in China (PPSV-23: 220-280 RMB [US $30.4-$38.7] per dose), leading to suboptimal uptake rates among older adults. Only 1% to 42% of older adults in China have received the pneumococcal vaccine [16]. This low uptake of PPSV-23 in China can be attributed to various factors, including financial constraints [17], vaccine hesitancy [18], and poor understanding [19]. Given these challenges, it is imperative to devise and implement innovative strategies to increase PPSV-23 vaccination among older adults.

Pay-it-forward is a socially innovative intervention that promotes health care service use [20]. With pay-it-forward, individuals receive a gift, such as a free or subsidized health care service. They are then invited to donate money or create a postcard with an encouraging message to support subsequent health care service recipients [21]. This intervention may help alleviate financial barriers that hinder the uptake of the PPSV-23. This intervention strategy may also promote positive health outcomes by improving vaccine trust and confidence [22]. Our previous studies have suggested that pay-it-forward interventions significantly increased influenza vaccination among older adults [23] and improved human papillomavirus vaccination among adolescent girls aged 15 to 18 years [24].

However, existing research on pay-it-forward mainly focused on more high-income cities [23,25,26]. Furthermore, no research has focused on using pay-it-forward to promote pneumococcal vaccination. This underscores the importance of exploring pay-it-forward as a strategy to boost PPSV-23 vaccination in resource-limited areas.

Guangxi is recognized as one of the resource-limited provinces in China. In 2022, the per capita disposable income of households in 94 of 111 counties in Guangxi was lower than the national average (US $5091.7) [27,28]. The pay-it-forward intervention may present a potential solution to increase the PPSV-23 vaccination rate among older adults in Guangxi.

### Aims and Hypotheses

This study will use a randomized controlled trial design with the primary objective of evaluating the effectiveness of the pay-it-forward intervention in promoting PPSV-23 vaccination in resource-limited settings. Through this research, we aim to compare vaccination uptake rates between the pay-it-forward and standard-of-care groups, and further assess the cost-effectiveness and per capita costs of both approaches. The findings will provide an innovative intervention model for improving pneumococcal vaccination among older adults in resource-ed-limited areas, offering critical evidence to inform government decisions on vaccine subsidy policies. Our hypotheses are as follows:

1. Pay-it-forward is superior to the standard-of-care in promoting pneumococcal vaccination.

2. The pay-it-forward intervention is associated with lower per capita costs of PPSV-23 vaccination.

## Methods

### Study Design and Setting

**Figure 1:**
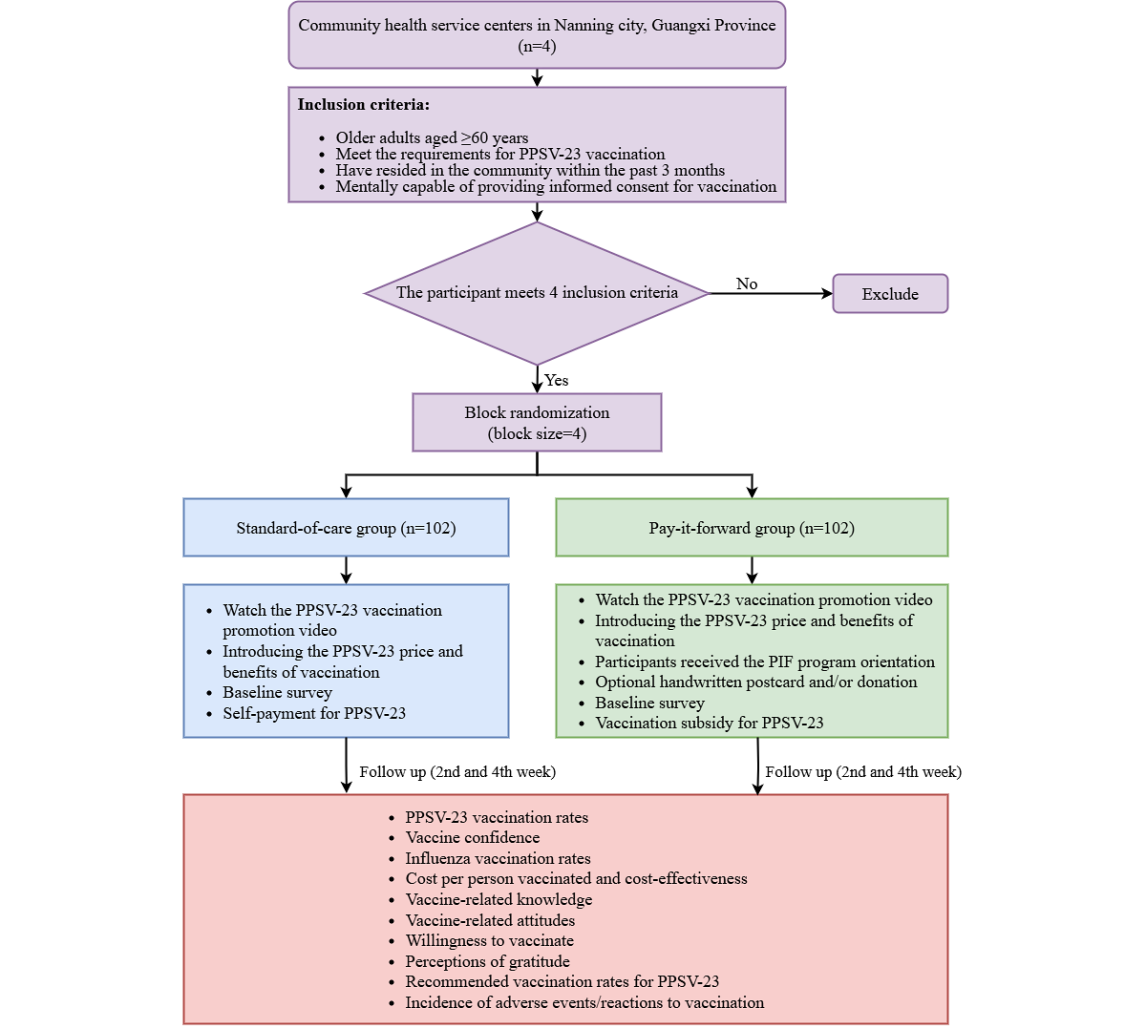
Framework diagram of the study. PIF: pay-it-forward; PPSV-23: 23-valent pneumococcal polysaccharide vaccine.

We will conduct a 2-arm, parallel randomized controlled trial among adults aged ≥60 years. Community health care centers (CHCs) will be the unit of randomization and intervention. We will recruit participants via vaccination clinics, WeChat (Tencent Technology Company Limited), and SMS text messaging. Participants will be randomly allocated to either the standard-of-care arm (self-payment for vaccinations) or the pay-it-forward arm (receiving a 150 RMB [US $20.7] subsidy for vaccination and opportunities for donation and handwritten postcards) in a 1:1 ratio through an envelope randomization mechanism, with blocks of 4 participants per group. Follow-ups are planned for the second and fourth weeks after enrollment of participants. At the end of the fourth-week follow-up, we will confirm the number of individuals vaccinated with the PPSV-23 in both groups through the vaccination information management system for the immunization program. Written informed consent will be obtained from each participant before enrollment. The CHCs are the central vaccination units for residents and have sufficient vaccine supplies and medical staff familiar with vaccination procedures, facilitating the recruitment of study participants. To ensure sample representativeness, we plan to conduct this study in Qingxiu district (high-income areas), Xixiangtang district (middle-income area), and Liangqing district (low-income area) in Nanning city, Guangxi province. All research assistants and community health care workers (CHWs) involved in this study will receive the same training. Figure 1 and Table 1 illustrate the study design and interventions.

**Table 1:** Schedule of events of the pay-it-forward for 23-valent pneumococcal polysaccharide vaccine (PPSV-23) study.

Event			Eligibility screening		Enrollment		Follow-up				Closeout
			Week 0		Week 0 (baseline)		Week 2		Week 4		Week 5
**Recruitment and enrollment**											
	Vaccination platform screening	✓									
	Phone or WeChat consultation	✓									
	Recruitment poster			✓							
**Intervention**											
	Pay-it-forward program leaflet			✓							
	PPSV-23 vaccination leaflet			✓							
	PPSV-23 promotional video			✓							
	Informed consent			✓							
	Group allocation			✓							
	Donation			✓							
	Handwritten postcard			✓							
	Baseline questionnaire			✓							
	Follow-up questionnaire					✓		✓			
**Assessment**											
	PPSV-23 vaccination rate									✓	
	Vaccination attitudes									✓	
	Willingness to vaccinate									✓	
	Perceptions of gratitude									✓	
	Recommendation of PPSV-23 vaccination									✓	
	Incidence of adverse events or reactions to vaccination							✓			
	Cost-effectiveness										

### Public Involvement Framework and Participant Recruitment

The public will be involved in creating project materials, such as developing a video explaining the concept of pay-it-forward, co-designing postcards to promote PPSV-23 vaccination [23], and discussing how to describe the pay-it-forward intervention to the participants.

To achieve the target sample size within the planned recruitment period, we will adopt multiple proactive recruitment strategies. Eligible participants will be identified and approached through vaccination clinics at participating CHCs, where staff routinely engage with this population. In addition, digital outreach strategies will be used, including invitations via WeChat messages and SMS text messaging notifications sent through local public health networks. The CHC staff will be trained to provide clear, culturally appropriate explanations of the study’s purpose and procedures to encourage participation. The intervention itself—including a 150 RMB (US $20.7) vaccination subsidy and the opportunity to write and receive handwritten postcards—is expected to enhance engagement and reduce financial and psychological barriers to participation. Recruitment progress will be monitored weekly by the coordinating research team. If enrollment falls behind schedule at any site, responsive strategies will include increasing outreach messaging, extending clinic hours, or deploying additional staff to support recruitment efforts.

### Community Engagement

To determine the effectiveness of the PPSV-23 and the benefits of vaccination for community residents, we will cooperate with local CHCs. CHWs will participate in the design of the pneumonia leaflet, refinement of the questionnaire, development of communication strategies, and formulation of recommendations to enhance the engagement of older adults in this study. The participants will also be invited to write postcards for subsequent participants.

### Participants

Eligible participants will be screened for participation in the program with the help of CHC health care workers. Inclusion criteria are as follows: (1) aged ≥60 years; (2) meeting the medical requirements for PPSV-23 vaccination; (3) residing in the community for the past 3 months; and (4) mentally capable of providing informed consent for vaccination. The participants will be excluded if they meet any of the following criteria: (1) older adults having severe chronic or psychiatric illnesses; (2) older adults having a clinically evaluated risk of allergy to PPSV-23; and (3) older adults having received PPSV-23 within the past 5 years.

### Randomization

This study will use block randomization to ensure balance between the intervention and control groups (block size=4). First, random sequences will be generated by computer from random seeds using SPSS software (version 25.0; IBM Corp). Each set of 4 random numbers will be grouped as a block and numbered in the order of generation. Subsequently, the random numbers within each block will be arranged in ascending order. The 2 smallest numbers will be assigned to the standard-of-care arm, and the 2 largest will be assigned to the pay-it-forward arm. Afterward, the study designer will write the corresponding numbers on the surface of opaque envelopes, insert the intervention material and group assignment information, and seal them. The participants will receive the envelopes corresponding to their assigned numbers and proceed with participation in the study.

### Blinding

Because both study researchers and participants are aware of the intervention allocation, achieving blinding poses a challenge. Nonetheless, several blinding techniques will be used to mitigate bias: (1) strict confidentiality regarding the generation and assignment of random numbers will be maintained among all the participants, research assistants, and CHWs, with only the study designer having access to this information; (2) the research assistants or CHWs responsible for allocating envelopes and interacting with participants will not be informed of the group information until the envelopes are opened; (3) the vaccine nurse will remain unaware of the grouping of participants; and (4) data analysts will be blinded of the intervention received by participants.

### Intervention

#### Pay-It-Forward Group

The research assistants at the vaccination clinics will use the PPSV-23 vaccination leaflet (Multimedia Appendix 1) to introduce participants in the pay-it-forward group to *S* *pneumoniae*, the PPSV-23 vaccination, and its benefits. Subsequently, they will use the pay-it-forward program leaflet (Multimedia Appendix 2) to explain the purpose of the program, access to the PPSV-23 vaccination subsidy (US $20.7), and the opportunities to donate and write a postcard encouraging subsequent pay-it-forward recipients to receive the PPSV-23. The participants in this group will be informed of the standard cost of the PPSV-23 vaccination (US $29.5-$30) and the donations made by previous pay-it-forward recipients, as well as the handwritten postcards containing encouraging messages for the PPSV-23 vaccination. After watching the PPSV-23 promotional video, the participants will be surveyed by a research assistant using questionnaire A. They will be asked whether they are willing to be vaccinated and whether they are willing to make a donation or write a postcard with a message to encourage subsequent pay-it-forward recipients to become vaccinated with PPSV-23. The participants may donate at the vaccination clinics or online using their enrolled number (ie, the envelope number). They will also be informed that their decision to donate and the amount donated will not affect their eligibility for the vaccine subsidy and access to PPSV-23. The participants who are willing to be vaccinated will be evaluated for vaccination eligibility and scheduled for an appointment. The donations received will be used to support subsequent pay-it-forward recipients, and the amount and use of donations will be regularly updated on the official WeChat account. The CHWs will conduct follow-up surveys using questionnaire B and questionnaire C during the second and fourth weeks after enrollment. Each participant will receive 100 RMB (US $13.8) for completing the 3 surveys. The intervention fidelity will be monitored using a standardized fidelity checklist (Multimedia Appendix 3).

#### Standard-of-Care Group

The participants in this group will also receive the same PPSV-23 vaccination leaflet, a promotional video, and a questionnaire (not including questions on pay-it-forward) similar to that provided to the pay-it-forward group. They will also be informed of the standard cost of the PPSV-23 vaccination (US $29.5-$30). After watching the PPSV-23 promotional video, the participants will be surveyed by a research assistant using questionnaire A and informed that they will need to pay for the vaccination at the standard market price. Participants who are willing to be vaccinated will be assessed for eligibility and scheduled for an appointment for vaccination. The CHW will also follow up with the participants in the second and fourth weeks after enrollment. The participants will receive 100 RMB (US $13.8) for completing the 3 surveys. The participants in the standard-of-care arm will not receive any information about pay-it-forward.

### Sample Size

The sample size was calculated using the PASS 2021 software after conducting a pilot trial. In the pilot trial, 88% (23/26) of the participants in the pay-it-forward group and 12% (3/26) of the participants in the standard-of-care group received the pneumonia vaccine. On the basis of the pilot trial results, we initially calculated the sample size to be 16. However, this sample size was determined to be too small to detect the desired effect size for the trial. To address this, we reviewed previous data on vaccination rates and estimated the expected difference in rates between the 2 groups based on prior experience. An earlier review indicated that the vaccination coverage of PPSV-23 among older adults in China ranged from approximately 1.23% to 42.10% [16]. Our previous pilot study showed that the pay-it-forward intervention increased influenza vaccination coverage in older adults by approximately 30% [23]. Considering these, we estimated a PPSV-23 vaccination coverage of 10% in older adults, and projected that the pay-it-forward intervention would increase this by 20%. The power of the test was set as 90% using a 2-sided test with a significance level of.05. The minimum sample size was calculated to be 158. After accounting for a 20% dropout rate, the minimum sample size required was adjusted to 198. The minimum sample size was further increased to 204 to accommodate the block-randomized design. Consequently, at least 204 participants were required for the trial.

### Data Collection and Management

When participants are enrolled, the research assistants will record their enrollment time, envelope number, name, identification number, and group. Data will be collected on the professional Chinese questionnaire website Wenjuanxing [29] using self-designed questionnaires, including the baseline questionnaire (questionnaire A) and the follow-up questionnaires (questionnaires B and C). The information collected is presented in Table 2.

**Table 2:** Summary of information to be collected.

Item	Questionnaire codes
Sociodemographics	A
Knowledge about PPSV-23^a^	A, B, and C
Dissemination of PPSV-23	A
Attitudes toward PPSV-23	A, B, and C
Gratitude (GQ-6^b^ scale)	A, B, and C
Willingness and reasons for receiving the PPSV-23	A
Feasibility, appropriateness, and acceptability of pay-it-forward (only pay-it-forward group)	A
Reasons for promoting or discouraging donations (only pay-it-forward group)	B and C
Incidence of adverse reactions or adverse events	B and C
Recommendation of PPSV-23 to others	B and C

^a^PPSV-23: 23-valent pneumococcal polysaccharide vaccine.

^b^GQ-6: Gratitude Questionnaire—6-item scale.

We will collect the following information: (1) sociodemographic characteristics (eg, sex, marital status, education levels, monthly income, and employment); (2) knowledge of PPSV-23 (eg, age eligibility, reduced risk of hospitalization and death, and reduced medical costs owing to pneumonia); (3) attitudes toward PPSV-23, which will be evaluated using a standardized scale [30] to assess vaccination confidence among participants (eg, perceived safety, efficacy, and importance of the vaccine); (4) education and sensitization related to PPSV-23 (eg, recommendations from health care workers or friends); (5) tendency to show gratitude, which will be measured using the widely used Gratitude Questionnaire—6-item scale (GQ-6) [30]. (6) reasons for promoting or discouraging donations, which will be assessed among participants in the pay-it-forward group, along with the feasibility, appropriateness, and acceptability of the pay-it-forward interventions, using previously well-evaluated scales [31]. (7) incidence of adverse reactions (eg, fever, redness, swelling, pain, itching) or adverse events (eg, pneumonia, bronchitis, ear infections), which will be investigated for participants who receive vaccination; (8) reasons for declining vaccination, which will be collected from the participants who do not receive the vaccine; and (9) participants recommendations to others to receive PPSV-23.

The uptake of PPSV-23 will be verified using the immunization program’s information management system. Separate questionnaires will be administered to participants in the standard-of-care and pay-it-forward groups. Quality control questions have been included to ensure the accuracy of questionnaire completion. Two similar questions will be included in different sections of the questionnaire to assess the consistency of responses: (1) Does vaccination with PPSV-23 reduce hospitalization and deaths? and (2) Vaccination with PPSV-23 reduces hospitalization and death. If participants respond differently to these 2 similar questions, the questionnaire will be considered invalid, and they will be asked to complete it again. Once participants are enrolled, personally identifiable information and survey data will be entered into an electronic Microsoft Excel form. The form will be password-protected by the study designer, and only the study designer and the researcher responsible for data analysis will have access to it. A data safety and oversight committee will be established, comprising 2 clinicians, an epidemiologist and statistician, and a representative of the medical ethics committee, who will operate independently of the research team. The data safety and oversight committee will review the process of the study, data collection, data management, and analysis. Each participant will be assisted by a CHW in completing the survey. For older adults who do not have a smartphone, the survey will be completed using an electronic device provided by the research program, and the CHW will guide them through the QR code scanning and submission process.

### Outcome Measures

The primary outcome of this study will be the uptake of PPSV-23 in each arm, assessed 1 month after the intervention using administrative records. The secondary outcomes will include: (1) vaccine confidence, measured by 4 survey statements related to perceptions of the importance, safety, and efficacy of vaccination, as well as vaccine management; (2) influenza vaccination, defined as participants receiving the influenza vaccine after enrollment in the study, verified through administrative records, with the cost of the vaccination borne by the participants; (3) cost per person vaccinated and cost-effectiveness; (4) vaccine knowledge between the 2 groups; (5) attitudes toward vaccination; (6) willingness to vaccinate; (7) gratitude, measured by the GQ-6 scale; (8) the rate of successful vaccine referral, defined as participants successfully recommending PPSV-23 to others in the community after participating in the study; and (9) incidence of adverse events or reactions to vaccination.

### Statistical Analysis

#### Primary Outcome

Descriptive analysis will be used to summarize the sociodemographic characteristics, knowledge, attitudes, and behaviors of the participants in each arm. Cronbach α will be used to test the reliability of the scales in the questionnaire. For the primary analysis, we will conduct intention-to-treat and per-protocol analyses, using logistic regression to compare the odds ratios of vaccination rates between the pay-it-forward group and the standard-of-care group, with adjustment for sociodemographic characteristics (eg, sex, age, and education level). In addition, we will perform logistic regression analyses stratified by income level, chronic disease status, living status, and distance to the vaccination site.

#### Secondary Outcomes

The economic evaluation will be conducted from a health care provider’s perspective with a 1-year time horizon. Microcosting methods will be used to evaluate the implementation costs of both the standard-of-care and pay-it-forward intervention groups. Specifically, we will collect cost data, including personnel time, medical supplies, equipment use, and facility expenses. Material procurement costs will be calculated using primary sources, such as invoices, while project implementation costs will be based on researchers’ self-reported data. Health care personnel’s time costs will be estimated using the local government–published wage standards. A decision tree model will be developed using TreeAge Pro 2022 software (TreeAge Software, LLC), with vaccination uptake (defined as the number of vaccinated individuals per intervention arm) serving as the effectiveness measure for the pay-it-forward intervention. The intervention will be compared against standard-of-care (participants’ self-paid PPSV-23 vaccination) to estimate incremental costs and incremental cost-effectiveness ratios. Model robustness will be assessed through 1-way sensitivity analyses and probabilistic sensitivity analysis. Influenza vaccination rates and vaccine confidence will be compared between the pay-it-forward intervention group and the standard-of-care group using logistic regression analyses, adjusted for sociodemographic information. Other secondary outcomes will be summarized using descriptive statistics and compared between the 2 arms.

Participants with missing primary outcome data (ie, vaccination status) will be reviewed. A complete case analysis will be performed if the proportion of missing primary outcome data is low (<5%). If the missing data exceed this threshold, multiple imputation methods will be applied under the assumption that the data are missing at random, to account for potential bias. Sensitivity analyses will also be conducted to assess the robustness of the findings under different assumptions about the missing data. To address missing follow-up data, we will use the last observation carried forward method or a multiple imputation method for missing values for categorical and continuous variables.

The results of all logistic regression analyses will be reported as crude odds ratios and adjusted odds ratios, along with 95% CIs. All statistical tests will be 2-sided, with a significance level of *P*<.05.

### Ethical Considerations

The study was approved by the medical ethics committee of Guangxi Medical University (Ethics No. KY0261, 2023) in accordance with the Declaration of Helsinki. All participants will be required to provide written informed consent before participating in the study. Participants have the option to opt out at any time. All collected data will be anonymized and deidentified to ensure privacy and confidentiality protection. Informed consent covers secondary analysis without additional consent. Participants who complete all three questionnaires will receive compensation of 100 RMB (US $13.99).

## Results

Participant recruitment took place from January 2024 to September 2024. We enrolled 220 participants. Final results are anticipated in June 2026.

## Discussion

### Expected Outcomes

The purpose of this study is to evaluate the effectiveness of a pay-it-forward intervention in promoting PPSV-23 vaccination among older adults aged ≥60 years. The results will inform the future development of pneumococcal vaccination interventions for older adults and contribute to the development of more cost-effective and sustainable public health policies.

The prevalence of pneumococcal diseases among older adults in China remains high, yet the coverage rate of PPSV-23 (ie, 1.23%-42.10%) [16] is significantly lower than that in high-income countries (52%-75%) [32-34]. Free pneumococcal vaccinations have been implemented for community residents in some high-income cities, such as Beijing, Shanghai, and Guangzhou [35]. However, in areas with limited health resources, the government faces challenges in providing free pneumococcal vaccines to community dwellers because of insufficient funding. Therefore, it is imperative to implement practical and feasible interventions to promote vaccination uptake. Previous studies have demonstrated the effectiveness of the pay-it-forward intervention targeting influenza vaccination or testing for gonorrhea and chlamydia in high-income areas of China [20,23]. However, the efficacy of this intervention in resource-limited regions has not yet been studied. This trial will be the first to assess the effectiveness of a pay-it-forward intervention in promoting the uptake of PPSV-23 for older adults in low-income areas in China. In addition, a self-designed questionnaire will be used to determine the differences in cost-effectiveness, knowledge, attitudes, willingness to vaccinate, and perceptions of gratitude between the pay-it-forward and standard-of-care groups. This study may provide insights into the development and optimization of pneumonia immunization strategies in regions with limited resources.

We anticipate that this study will provide evidence supporting the feasibility of the “pay-it-forward” intervention in vaccine services and offer valuable insight for evidence-based practice in future vaccine programs. Currently, the PPSV-23 is not included in China’s National Immunization Program, requiring individuals to bear the cost of vaccination, which limits the uptake of the PPSV-23 in resource-limited areas. The “pay-it-forward” approach may promote vaccination in these areas. In the absence of government funding for vaccination, the pay-it-forward model may effectively pool small donations to support more individuals in accessing pneumonia vaccine services, which is of significant importance for improving vaccination coverage in areas with relatively insufficient health resources. Moreover, the “pay-it-forward” strategy has demonstrated high cost-effectiveness in promoting influenza vaccination [36]. By evaluating cost-effectiveness, this study may inform decision-making regarding expansion strategies, enabling stakeholders to make informed resource allocation decisions.

Previous studies have shown that implementing free vaccination policies has enhanced the coverage of PPSV-23 [37,38]. However, implementing free vaccination strategies in low-income areas with limited health resources remains challenging. Prior randomized controlled trials have explored educational information interventions to increase vaccination uptake [39,40]. However, immediate improvements in knowledge and risk perception have not immediately led to changes in vaccination behavior. Vaccine price, vaccine accessibility, and vaccine confidence are additional factors influencing vaccination behavior. This study is the first to use a randomized controlled trial to evaluate the effectiveness of the pay-it-forward intervention for PPSV-23 vaccination. Through community engagement, vaccination subsidies, and public education, the intervention aims to enhance knowledge about the vaccine, bolster confidence in its safety and efficacy, attract financial contributions, and ultimately promote vaccination.

### Strengths and Limitations

This study uses a randomized controlled trial to evaluate the effectiveness of the innovative pay-it-forward intervention in improving PPSV-23 vaccination rates among older adults, while comprehensively assessing its multidimensional impacts on vaccination-related cognition, behaviors, and economic outcomes. By validating this novel approach that integrates economic incentives with social mobilization, the findings will provide evidence-based insights for developing cost-effective vaccination promotion strategies, particularly facilitating the optimization of pneumococcal vaccination policies in low- and middle-income regions.

Some limitations exist in this study. First, the questionnaires rely on self-reports, which may introduce biases in specific responses. However, this should not impact the vaccination rate, as it will be verified through the vaccination information management system of CHCs. Second, the sample size for exploring factors associated with willingness to vaccinate is insufficient, and additional cross-sectional studies with rigorous sampling and a larger sample size are necessary to determine these factors. Third, conducting 2 follow-up surveys within a month may improve the participants’ knowledge of vaccines to some extent, but it may not be sufficient to change their attitudes and behaviors toward the PPSV-23 quickly. Finally, there is a possibility of cross-contamination in this study. We implemented contamination control measures: (1) prohibiting the sharing of intervention details; (2) using group-specific materials and separate discussion spaces; and (3) applying both intention-to-treat and per-protocol analyses to assess potential contamination effects.

### Future Directions

Future research should incorporate a free vaccination group to better isolate and quantify the specific effects of the pay-it-forward intervention. This innovative model is expected to facilitate the uptake of public health services, and additional implementation studies (eg, disease screening) could be conducted to assess its feasibility in different settings.

### Dissemination Plan

The trial results will be disseminated to the study participants, health care professionals, public health authorities, and the scientific community. The findings will be published in peer-reviewed journals, presented at conferences, and reported in trial registries, such as the Chinese Clinical Trial Registry (ChiCTR). Briefings will be provided to participating CHCs, and lay summaries may be shared with the public via digital platforms (eg, WeChat).

### Conclusions

In summary, this study aims to assess the effectiveness and cost-effectiveness of the “pay-it-forward” intervention in the vaccination with PPSV-23 among older adults. This innovative payment model may offer a new approach to enhance vaccination uptake in resource-limited regions, ensuring access to vaccination services, especially in settings with limited health care resources. In addition, the pay-it-forward strategy may act as a transitional step toward gradually shifting from individual payment-based vaccination policies to publicly funded ones.

## Appendix

Multimedia Appendix 1 Vaccination leaflet of 23-valent pneumococcal polysaccharide vaccine.

Multimedia Appendix 2 Pay-it-forward program leaflet.

Multimedia Appendix 3 Fidelity checklist of pay-it-forward intervention.

## Abbreviations

CHC: community health care center
CHW: community health care workers
GQ-6: Gratitude Questionnaire—6-item scale
PPSV-23: 23-valent pneumococcal polysaccharide vaccine

## Data Availability

Data sharing is not applicable to this article as no datasets were generated or analyzed during this study. The datasets generated or analyzed during this study will be available from the corresponding author on reasonable request.
