# Supplementary material for: Pay-It-Forward 23-Valent Pneumococcal Polysaccharide Vaccination Among Older Adults: Protocol for a Randomized Controlled Trial
Source: JMIR Res Protoc. 2025 Sep 8;14:e70246. doi: 10.2196/70246 (PMC12455148; doi:10.2196/70246)
Supplement: Multimedia Appendix 2 [file resprot_v14i1e70246_app2.pdf]

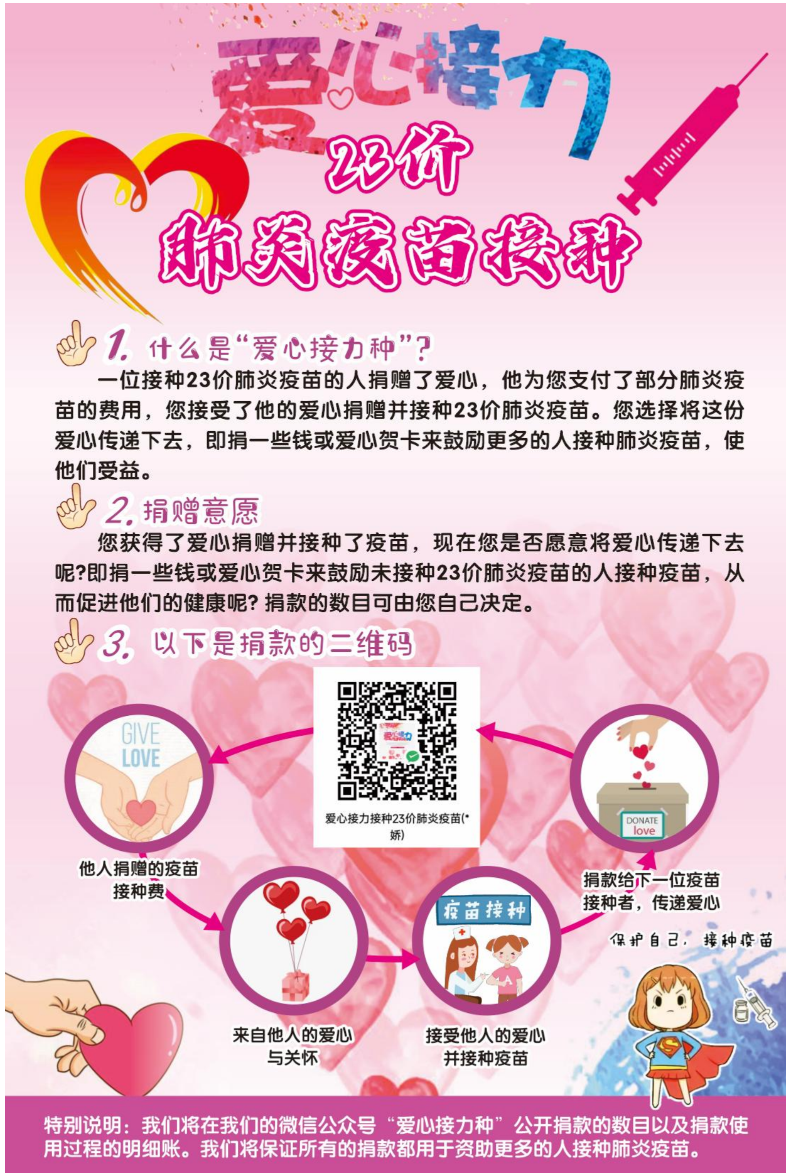

Pay-it-forward intervention to promote 23-valent pneumococcal polysaccharide vaccination

1. What is the “pay-it-forward”?
A person who got the 23-valent pneumococcal polysaccharide vaccine made a donation, he paid part of the cost of the vaccine for you, and you accepted his donation and got the 23-valent pneumococcal polysaccharide vaccine. You choose to pass on the love, i.e. donate some money or write a postcard to encourage more people to get the 3-valent pneumococcal polysaccharide vaccine so that they can benefit from it.

2. Willingness to Donate
You have been donated and vaccinated, now would you like to pass on the love? That is, donate some money or write a postcard to encourage people who have not got the 23-valent pneumococcal polysaccharide vaccine to get vaccinated and thus promote their health. You decide how much you want to donate.

3. QR code for donations
(1) Vaccination costs donated by others
(2) Love and care from others
(3) Receive love from others and get vaccinated
(4) Donate to the next vaccine recipient and pass on the love

Note: We will share the number of donations and the details of how the donations are used on our public WeChat website "pay-it-forward to promote 23-valent pneumococcal polysaccharide vaccination". We will ensure that all donations will be used to support more people to get vaccinated against pneumonia.
