# Supplementary material for: Pay-It-Forward 23-Valent Pneumococcal Polysaccharide Vaccination Among Older Adults: Protocol for a Randomized Controlled Trial
Source: JMIR Res Protoc. 2025 Sep 8;14:e70246. doi: 10.2196/70246 (PMC12455148; doi:10.2196/70246)
Supplement: Multimedia Appendix 3 [file resprot_v14i1e70246_app3.docx]

# Multimedia Appendix 3

# Fidelity Checklist for the Pay-it-Forward Intervention Across Sites

| Item No. | Checklist Item | Completion (✓/✗/~) | Notes |
| --- | --- | --- | --- |
| 1 | Intervention staff received standardized training, including the pay-it-forward concept and communication procedures. |  |  |
| 2 | Standardized intervention materials (e.g., brochures, posters, videos, postcards) were used. |  |  |
| 3 | The pay-it-forward model was clearly and accurately explained to each participant. |  |  |
| 4 | Participants were informed that vaccination was voluntary and independent of donation decisions. |  |  |
| 5 | Participants were invited to voluntarily make a donation decision after vaccination. |  |  |
| 6 | Participants were informed that donations were voluntary and that contributions would be disclosed anonymously. |  |  |
| 7 | Each participant’s donation decision (donate / not donate / undecided) was recorded. |  |  |
| 8 | An anonymous donation option (e.g., donation box, QR code) was provided onsite. |  |  |
| 9 | Vaccination and subsequent data recording (e.g., consent forms, vaccination records) were completed according to the standardized procedure. |  |  |
| 10 | Independent quality control staff conducted regular onsite monitoring during intervention delivery. |  |  |
| 11 | Major deviations or unexpected events during intervention delivery were recorded and reported. |  |  |

Note: This fidelity checklist was developed based on the intervention design of this study to assess the consistency of pay-it-forward intervention delivery across study sites. Symbols: ✓ indicates completed; ✗ indicates not completed; ~ indicates partially completed with minor deviations from the protocol
